# T3 levels and thyroid hormone signaling

**DOI:** 10.3389/fendo.2022.1044691

**Published:** 2022-10-27

**Authors:** Federico Salas-Lucia, Antonio C. Bianco

**Affiliations:** Section of Adult and Pediatric Endocrinology and Metabolism, The University of Chicago, Chicago, IL, United States

**Keywords:** T3-signaling, deiodinase, thyroid, thyroxine, NTIS, thyroid hormone binding proteins, Triiodothyronine

## Abstract

The clinical availability of tissue-specific biomarkers of thyroid hormone (TH) action constitutes a “holy grail” for the field. Scientists have investigated several TH-dependent markers, including the tissue content of triiodothyronine (T3)—the active form of TH. The study of animal models and humans indicates that the T3 content varies among different tissues, mostly due to the presence of low-affinity, high-capacity cytoplasmic T3 binding proteins. Nonetheless, given that T3 levels in the plasma and tissues are in equilibrium, T3 signaling is defined by the intracellular free T3 levels. The available techniques to assess tissue T3 are invasive and not clinically applicable. However, the tracer kinetic studies revealed that serum T3 levels can accurately predict tissue T3 content and T3 signaling in most tissues, except for the brain and pituitary gland. This is true not only for normal individuals but also for patients with hypo or hyperthyroidism–but not for patients with non-thyroidal illness syndrome. Given this direct relationship between serum and tissue T3 contents and T3 signaling in most tissues, clinicians managing patients with hypothyroidism could refocus attention on monitoring serum T3 levels. Future clinical trials should aim at correlating clinical outcomes with serum T3 levels.

## Introduction

Why study tissue T3 levels? T3 is the physiologically active thyroid hormone (TH). Hence, all things being equal, fluctuations in the tissue T3 content are likely to reflect T3 action in any given tissue. Knowing the tissue T3 content is a good starting point for understanding T3 action (with the caveat that only the T3 physically bound to TH receptors (TRs) can trigger biological effects). Furthermore, a better understanding of tissue T3 content could give us an enhanced view of TH action in several clinical conditions that we currently only assume we understand. For example, how good a job are we doing in restoring TH economy and T3 action while treating patients with hypothyroidism? Is it possible that patients that experience residual symptoms have lower tissue T3 levels? How compromised is TH action (if at all) during caloric restriction or in patients with non-thyroidal illness syndrome (NTIS)? Unfortunately, the techniques available to study tissue T3 content are invasive and not clinically applicable. Thus, we still need to rely on serum T3 levels to infer T3 action. Here we reviewed the literature on tissue T3 content in animal models and humans, as well as the role played by deiodinases in regulating tissue T3, with emphasis on hypothyroidism. Other excellent reviews have focused on the role played by TH transporters and TRs (1–3).

Most T4 and T3 circulate in the blood tightly bound to carrier proteins—thyroxine (T4)-binding globulin or transthyretin—which act as a circulating reservoir of TH not directly available for cellular uptake (4). Only the much less abundant unbound (free) T4 and T3 molecules enter tissues and cells through specific transporters to form an intracellular pool of TH. These transporters are plentiful and generally expressed in abundance, exhibiting a great deal of redundancy. The brain, however, is an exception. In rare cases, inactivating mutations in the X-linked monocarboxylate transporter 8 (MCT8) TH transporter can lead to severe brain hypothyroidism. A malfunction in MCT8 deprives neural cells of sufficient amounts of TH without an alternative pathway, resulting in a complex neurological phenotype—the Allan-Herndon-Dudley syndrome—with devastating consequences. A study analyzing brain sections from MCT8-deficient fetuses identified a 50% reduction in T3 and T4 and reverse T3 contents in the cerebral cortex but normal T3 and T4 contents in the liver (5). Accordingly, studies of the MCT8 deficient mouse (MCT8-KO) show that different tissues exhibit variable MCT8 dependability. For instance, the MCT8-KO liver (that expresses other transporters (6)), has the expected content of T3 (based on the higher serum T3 levels). In contrast, tissues such as the brain, with limited redundancy in TH transporters (2), exhibit reduced T3 content (7). Rare inactivating mutations of TRs, TRα or TRβ, can also lead to clinically relevant disruptions of TH signaling (3).

According to the estimated volume of distribution for each hormone, 2/3 of the T3 remains inside tissues whereas 2/3 of T4 remains in the plasma (8). Because the plasma and tissue compartments are in equilibrium —the amount of T3 entering and exiting the tissues is the same—any changes in plasma T3 levels will directly impact the tissue content of T3. As an example, imagine two different size swimming pools, A and B, connected through a channel. Any changes in the water level in pool A will directly affect pool B and vice versa, regardless of the sizes of the pools. Because both pools are in equilibrium, one can infer the water level of pool A by measuring the level of pool B and vice versa.

Once inside cells, T3 may bind to cytoplasmic proteins, which are known for having high capacity but low affinity for T3 (9). Nonetheless, similar to the situation with the plasma binding proteins, there is a cytosolic pool of free T3, and only free T3 molecules are available for entering the cell nucleus and binding to TRs, which are low-capacity high-affinity proteins (9, 10). T3 binds to TRs with an affinity ~20-fold higher than T4, albeit there is evidence that T4 could trigger limited genomic (11) and non-genomic effects as well (12). Not much is known about how T3 enters the cell nucleus. It has been proposed that a nuclear/cytoplasm gradient of T3 exists but direct experimental data supporting this assumption is missing (10). At the same time, T3 may bind cytoplasmic TRs and accelerate their translocation to the cell nucleus (13).

Therefore, the variable presence of these high-capacity low-affinity cytoplasmic T3-binding proteins invalidates the assumption that the higher the tissue T3 content, the greater the magnitude of the T3-signaling in that tissue. Tissues with higher levels of binding proteins will have higher T3 content, which does not necessarily translate into enhanced T3 signaling—only the free T3 can enter the cell nucleus and bind to TR. While comparing T3 content among tissues is not very informative, when the same tissue is considered, tissue T3 content can be used as an index of T3-signaling.

Two T3-binding cytoplasmic proteins (in rodents) are unusual because they have a much higher affinity for T3, close to the one exhibited by TRs. These are µ-crystallin (CRYM) in the liver (14) and the nicotinamide adenine dinucleotide phosphate (NADPH)-dependent cytosolic T3-binding protein (CTBP) (15) in the kidney. Hence, these organs are examples of tissues in which TH levels are relatively high (due to the presence of these proteins) (16). It is not clear whether these specific proteins simply function as a reservoir of T3 or do play a role in TH signaling. In humans, at least one very rare CRYM mutation that eliminates binding to T3 is associated with deafness (17–19). But a causal-effect relationship has not been established particularly in the light that a CRYM-deficient mouse has normal hearing, despite a 20–50% reduction in T3 content in the brain, heart, liver, and kidney, without changes in serum T3 levels (16).

## Measuring T3 content in tissues

Assessing TH action within a tissue or organ by measuring tissue T3 content makes sense. This was first done by constantly infusing rats with ^125^I for weeks until an isotopic equilibrium—between ^125^I and the endogenous natural iodine—had been reached. Rats were then killed, and the content of tracer molecules in the plasma and each tissue was measured through paper chromatography (20). By determining the isotopic plasma/tissue ratio of T3, one could then estimate the gravimetric amounts of T3 in the tissue. Later, this method was much improved with the injection or infusion of radiolabeled T3 molecules (21).

The availability of specific radioimmunoassay (RIA) for T3 opened new possibilities, and direct measurements of T3 in rat serum and tissues were soon described (22). The key aspect of this approach is to correct for the efficiency of the T3 extraction, which is done by adding tracer amounts of radiolabeled T3 to the tissue homogenate. The gravimetric content of T3 in the tissue is subsequently measured by RIA. More recently, high-performance liquid chromatography (HPLC) coupled with mass spectrometry (MS) has been used as a highly specific and sensitive technique to assay T3 after tissue extraction. First validated in rat plasma and tissues (23), followed by human plasma (24), and later applied to tissue homogenates, this technique allows for the assay of tissue T3 in samples weighing only 50–300 mg (25–28).

A compilation of studies that analyzed the T3 content in tissues of rodents (20, 21, 26, 29–31) and sheep (32), showed similar levels in most tissues, with some notable exceptions. The organs with the highest T3 content (ng/g wet weight) are the kidney (6.6 ± 1.7), the anterior pituitary gland (5.7 ± 1.3), and the liver (4.5 ± 1.0), followed by the brain (2.8 ± 0.7), and lung (2.3 ± 0.9). Other tissues, such as the stomach and the prostate, presented lower T3 content (1.1 ± 0.3). It is notable that in rodents, the tissue T3 concentration greatly exceeds the levels present in the plasma (0.44 ± 0.05).

Studies to determine T3 content in human tissues are limited by the invasive nature of the methods available. Nonetheless, the available data suggests that no major differences exist between rodents and humans and that in humans too there is heterogeneity among tissues. A comprehensive study of embryonic specimens (abortions between 6-12 weeks), fetal and neonatal specimens (abortion or stillbirth between 15-36 weeks), and adult specimens from men (after death for cardiovascular diseases; 45-65 years) (22), used separation by column chromatography followed by RIA to measure tissue T3 content. In embryonic tissues, the highest T3 content was found in the brain, with similar figures also reported in 10-18 week fetuses (33). In fetuses and neonates, the liver, followed by the brain, tongue, muscle, and heart, were the only organs with a T3 content higher than in blood; lower values were found in the kidney and lungs.

In adult humans, the tissues with higher T3 content were the liver, brain, and kidneys, trailed by the heart, lung, stomach, spleen, and skin; T3 contents in the tongue and cartilage were undetectable. In another study, tissues from ~30-year-old males and females who died suddenly (34, 35) revealed that the kidney, followed by the anterior pituitary, hypothalamus, and liver, were the only organs with a T3 content higher than plasma. Lower values were found in the heart, skeletal muscle, cerebral cortex (prefrontal cortex (36)), and lung.

Overall, the available studies indicate that in humans, T3 content is highest in the liver, kidney, and brain. Like with rodents, this heterogeneous distribution does not reflect the intensity of the TH signaling but rather the existence of inherent tissue-specific properties that affect T3 content (e.g., the expression of low-affinity, high-capacity T3-binding proteins) without affecting T3 signaling.

## Tissue sources of T3

Some tissues express deiodinases, enzymes that can transform T4 to T3 (type 2 deiodinase, D2), T4 to rT3 (type 3 deiodinase, D3), or T3 to T2 (D3). These enzymes can also affect tissue T3 content, but independently of the plasma levels of TH (37, 38). Deiodinases are the main mechanism controlling TH signaling in the developing embryo (39, 40). Notably, the impact of each deiodinase on the local T3 content is different. D3 expression functions as a T3 sink, disrupting the equilibrium between plasma and tissue. Because of the D3 expression, the amount of T3 entering any given tissue will be greater than the amount of T3 exiting the tissue. Following the swimming pool analogy, the D3 expression is analogous to a leak in one of the pools. A clinical example of this phenomenon was provided by measuring the difference in plasma T3 levels between the aortic root (incoming blood) and the coronary sinus (outgoing blood) of patients with aortic stenosis undergoing cardiac surgery. The levels of T3 dropped significantly during myocardial perfusion because of the ectopic D3 expression in the hypertrophic myocardium (41). In contrast, D2 expression will increase tissue concentration of T3, and eventually, more T3 will exit the tissue when compared to what enters. In this case, D2’s role would be analogous to a hose dumping water into one of the pools.

While the methods to quantify tissue T3 are accurate and precise, most of them do not allow for the study of the role played by the deiodinases. In most tissues, the main (only) source of T3 is the plasma, but when present, D2 also constitutes a significant source of T3. To study this, the technique of isotopic equilibrium was modified to accommodate the simultaneous equilibration of T3 molecules labeled with either one of two distinct isotopes. Rats were injected with ^125^IT4 and ^131^IT3 at the appropriate times and killed once the equilibrium between plasma and tissues had been reached. The subsequent measurement of plasma and tissue ^125^IT3 and ^131^IT3 allowed for the quantification of the role played by D2 as a local source of T3 (37, 42, 43). In other studies, osmotic minipumps were used to deliver the radiolabeled TH molecules (44).

To avoid the interference created by the low-affinity high-capacity T3-binding cytoplasmic proteins, the studies that defined D2’s role in regulating TH signaling were based on the T3 content within the cell nucleus, with T3 content expressed in ng/mg DNA. Once the TR binding capacity, occupancy, and sources of T3 were determined for each tissue, it became clear that in most tissues, TR occupancy (with T3) fluctuates around 50% (e.g., liver and kidney), mostly with T3 derived from plasma. This means that based on the TR affinity for T3 (Ka= ~10^10^L/M) and the free T3 level in the plasma (~10pM; and supposedly cytosol), about half of all TRs are occupied with T3 at any given time. In contrast, TR occupancy with T3 is much higher in tissues expressing D2, about 90% in the brain, and 80% in the pituitary gland and brown adipose tissue (BAT) (43, 45). Furthermore, the activation of D2 increases even further the TR occupancy level with T3 (up to ~100%) with locally generated T3, while T3 levels in the circulation fluctuated minimally (37, 46). In all these tissues, most nuclear T3 was generated locally *via* the D2 activity. These data illustrate a phenomenal mechanism to regulate TH signaling and the difficulties in predicting T3 action in the brain, pituitary gland, or BAT based solely on plasma T3 levels.

In humans, evidence that D2 plays a role in defining tissue T3 content is indirect, based on the T3 content of the tissue and/or on physiological mechanisms. For example, the T3 content in different parts of the human brain at week 20 of gestation reaches higher levels than in adults, even though circulating T3 levels in the fetus are much lower. The cerebral cortex T3/T4 ratio increases throughout this period, in contrast to the plasma T3/T4 ratio, which tends to decrease (47). This suggests that the high D2 activity in the week 20 cerebral cortex and a very low cortex D3 activity play a role. At the same time, cerebellar T3 content is very low, which also exhibits the highest D3 activity in the developing brain. Other brain regions with high D3 activities (midbrain, basal ganglia, brain stem, spinal cord, and hippocampus) also exhibit low T3 content (47).

The functioning of the TSH/TRH feedback mechanism constitutes another powerful indirect evidence that D2 modulates T3 signaling in humans (42). In rodents, D2 is co-expressed in the TSH-secreting cells on the pituitary gland (48), and most (>50%) TRs in the pituitary are occupied with D2-generated T3 (42). The potential role played by D2 in humans is concluded by the fact that in normal adult individuals, serum TSH levels exhibit an inverse relationship with serum T4 levels, even as T3 levels remain relatively stable. This is illustrated in patients during the early phases of hypothyroidism or iodine deficiency, in whom a slight reduction in serum T4 is sufficient to elevate serum TSH, much before serum T3 levels have been affected (49, 50). Thus, it is very likely that in humans as well, the T3 content in TSH secreting cells is greatly influenced by the local D2 activity.

## Changes in tissue T3 content in disease states

Having clinically relevant tissue-specific biomarkers that reflect T3 action is the ultimate goal of clinical scientists that study TH action (51). For example, serum TSH, is the best biomarker for T3 action in the medial-basal hypothalamus and pituitary gland, reflecting the plasma levels of both T4 and T3. Other tissue-specific biomarkers of T3 action exist, which exhibit some tissue-specificity but lack sensitivity. For example, the basal metabolic rate reflects T3 action in metabolically relevant tissues, the serum cholesterol or SHGB levels, and the myocardial isometric contraction time, which reflects T3 action in the liver and heart, respectively. More recently, the utilization of single-cell RNA-seq data and proteomic tools have allowed for identifying cell types sensitive to TH regulation in tissues and accurate tissue-specific biomarkers of TH action that await clinical validation (52–54).

Measuring tissue T3 content remains an excellent tool to assess tissue-specific TH action while we wait for the development of clinically relevant biomarkers of T3 action. But how can this be accomplished in humans if the methods are too invasive and not practical? By measuring serum T3 levels. Undoubtedly, serum T3 levels can accurately predict the T3 content in multiple tissues, except the ones that express either D2 or D3. Indeed, experimental data in rats, for example, indicate that a 10% fluctuation in serum T3 levels will impact, with the same intensity, the T3 content and TH action in the liver (55).

As discussed above, the limited data available from donors and patients support that this is the case in humans as well. As a result, in a patient with hyperthyroidism, a 20% increase in serum T3 levels should be followed by an equivalent increase in the T3 content of most tissues and the TH signaling. Conversely, the opposite scenario takes place in patients exhibiting a drop in serum T3 levels. Thus, measuring serum T3 levels is useful to estimate T3 content and TH signaling in most tissues, and could be valuable in the management of patients with hypothyroidism.

Physicians have not frequently used serum T3 levels for the *diagnosis* of hypothyroidism. This is because of the homeostatic mechanisms (adjustments in thyroid secretion of T3 and deiodinase activity) that evolved to preserve plasma (and tissue) T3 levels within the normal range. These mechanisms are effective during iodine deficiency, or the initial phases of hypothyroidism as discussed above. Preserving T3 levels in both situations minimizes signs and symptoms of overt hypothyroidism. Thus, serum T3 levels fare poorly as a tool to diagnose hypothyroidism (when compared to serum FT4 or TSH). Nonetheless, monitoring serum T3 levels during the treatment of patients with hypothyroidism could be of value (see below).

### Hypothyroidism

Overtly hypothyroid rats exhibit a reduction in tissue T3 content (~90%), which is associated with a marked decrease in TH signaling (26, 56–59). This is restored upon treatment with T3, following a direct (non-linear) relationship between nuclear T3 content (TR occupancy) and TH signaling (55, 60, 61). Thus, given that the multiple compartments holding T3 (plasma, cytoplasm, and cell nucleus) are in equilibrium, a reduction in serum T3 levels predicts that tissue T3 and nuclear T3 contents are reduced at the target tissue level.

No data on tissue T3 content is available for patients with hypothyroidism, making it harder to translate the findings from rats to humans. Nonetheless, there is a clear and objective indication that plasma and tissue T3 contents are linked and that changes in plasma T3 are directly translated into changes in the T3 content of most tissues (59, 62).

This could have clinical consequences given that LT4-treated patients with normal serum TSH levels may not have fully restored TH economy. In these patients, serum T4 levels are slightly elevated (~10%) whereas serum T3 levels are slightly lowered (~10%). In about 15% of the patients, serum T3 levels are reduced below the normal reference range (63–65). Based on all that was discussed above, these data indicate that in a substantial number of LT4-treated patients, T3 serum and tissue contents as well as T3 signaling may have not been fully normalized.

Some clinical studies lend support to this line of reasoning. First and foremost, the rate of energy expenditure is not restored in LT4-treated patients despite the normalization of serum TSH (66–69). Second, LT4-treated patients with normal serum TSH weigh about 10 pounds more and exhibit higher levels of serum low-density lipoprotein and total cholesterol when compared to controls (70), despite exhibiting a higher utilization of statins (71). Is there an opportunity here for clinicians to refocus attention on serum T3 levels in the management of patients with hypothyroidism?

In addition to these residual metabolic abnormalities, the possibility that the relatively lower serum T3 levels contribute to the syndrome of residual symptoms observed in about 10-20% of the patients treated with LT4 has also been investigated. However, while logical, clinical trials failed to observe a robust correlation between lower serum T3 levels and residual symptoms of hypothyroidism (72, 73). In other words, the patients that exhibit these residual symptoms are not the ones that have the lowest serum T3 levels. This apparent conundrum is explained by the fact that plasma T3 levels are not good predictors of T3 content in the brain. Given that these residual symptoms are largely cognitive, the lack of correlation is understandable.

Seminal studies by Silva and Larsen in rats revealed that for the brain plasma T3 does not accurately predict tissue or nuclear T3 content; they linked this to the presence of D2 in the brain (74). They showed that during hypothyroidism, there is a compensatory acceleration in D2 activity that explains the faster T4 to T3 conversion. They concluded that the lower the serum T4 levels, the higher the D2 contribution to the circulating (and tissue) T3. The acceleration in D2 activity is so effective that it delays the drop in plasma and tissue T3 seen during hypothyroidism. Furthermore, a faster D2 preserves T3 content in the brain and BAT, even after plasma T3 levels have dropped. Later, it was confirmed that also in humans the conversion of T4 to T3 is accelerated during hypothyroidism, which also delays the drop in serum (and presumably tissue) T3 levels (75, 76). A corollary of these studies is that an adaptive mechanism exists in some tissues (linked to D2 expression) that defends T3 content against a drop in plasma T3.

Indeed, the role played by D2 in maintaining plasma and tissue T3 contents during the treatment of hypothyroidism was elegantly illustrated by Obregon and Morreale de Escobar (62). They revealed that treatment with LT4 has variable effectiveness in restoring T3 levels, depending on the tissue analyzed. It is very effective in tissues expressing D2, which includes the pituitary gland that secretes TSH. Thus, no single dose of LT4 given to rats with hypothyroidism could normalize TSH levels and T3 content in all tissues simultaneously (62). In some tissues, only supraphysiological plasma T4 could normalize T3 content, but at the expense of suppressing serum TSH levels. Subsequent studies using a similar rat model obtained direct evidence of tissue hypothyroidism in LT4-treated rats at doses that normalized TSH levels (77). In this case, a series of T3-dependent genes was studied in the liver, heart, and skeletal muscle. Further studies using combined replacement therapy with LT4 and LT3 did restore tissue T3 levels in thyroidectomized rats at much lower doses of T4 than those needed to normalize T3 in most tissues when T4 alone was used (78). Overall, only the combined treatment with LT4 and LT3 was able to restore tissue T3 content and TH signaling simultaneously with the normalization of serum TSH.

In rats, the cerebral cortex and cerebellum display a remarkable degree of independence from plasma T4 and T3 levels, presenting normal or near normal T3 content over a wide range of T4 doses. T3 homeostasis in these organs is independent not only of the T4 dose but also of plasma T4 and T3 levels and could be explained by the adaptive changes in D2 activity. With this in mind, how can we reconcile the effectiveness of these homeostatic mechanisms with the fact that some LT4-treated patients remain symptomatic with detriments of cognition, mood, and quality of life? (79).

Given the pivotal role played by local D2-mediated T4 to T3 conversion in the brain, is it possible that “defects” in D2 could compromise the effectiveness of therapy with LT4 in restoring T3 homeostasis in the brain? Within the brain, D2 is expressed in glial cells, astrocytes, and tanycytes, but not in neurons (80, 81). Astrocytes are intimately related to neurons (hundreds of thousands of neuronal synapses per astrocyte); an array of metabolites are known to be preferentially exchanged between astrocytes and neurons (82). Hence, the concept that the brain responsiveness to LT4 is mediated by astrocyte-derived T3 production and transfer to neighboring neurons is well accepted (83, 84). Accordingly, mice lacking D2 in the glial cells exhibit brain hypothyroidism with cognitive dysfunction (85).

A common polymorphism in the DIO2 gene (Thr92Ala-DIO2), which alters the intracellular localization of the enzyme and reduces D2 activity by ~20%, has been associated with impaired effectiveness of LT4 in the treatment of hypothyroidism, and clinical improvement when liothyronine (LT3) was added to LT4 therapy (73, 86–88). Although not universally observed (89), these findings highlight the potential for the D2 pathway to affect normal TH signaling in the brain and modify the effectiveness of therapy with LT4. Indeed, a mouse carrying the Thr92Ala-Dio2 polymorphism exhibits a transcriptome and a cognitive phenotype suggestive of a localized reduction in TH signaling in the brain–the mice also responded positively to therapy with LT3 (90).

### Non-thyroidal illness syndrome

Illnesses can induce major modifications in the TH economy, i.e., NTIS aka low T3 syndrome. NTIS patients show lower serum levels of T3, higher reverse T3, lower free T4, and inappropriately normal or slightly low serum TSH levels. However, the lack of clear signs and/or symptoms of hypothyroidism in these patients, questions whether T3 signaling (and/or tissue T3 content) is equally reduced (91).

Studies in rodent models with different severity levels of NTIS (in which serum T3 is reduced by ~2-3 fold) show that the T3 content of different tissues is not all equally affected. For example, the reduction in muscle T3 content is less severe than in the liver (~30 vs. 50%, respectively) (92). In addition, hypothalamic T3 content remained unaffected in a study using a rabbit model of prolonged NTIS, despite a ~50% drop in serum T3 (93).

Along the same lines, patients with severe NTIS may exhibit ~80% lower serum T3 levels as well as 40-75% lower T3 content in the cerebral cortex, hypothalamus, pituitary, liver, kidney, and lung. Notwithstanding, tissue T3 content in these patients’ hearts and skeletal muscles remained unaffected (34). In a study on NTIS patients that died, the decrease in serum T3 levels was also associated with a reduction in liver and muscle T3 contents (36). An additional complicating factor in NTIS patients is the ectopic expression of D3 in multiple tissues. The tissue-specific accelerated inactivation of T3 magnifies the drop in T3 content caused by the reduction in plasma T3 levels. Thus, it is difficult to predict tissue T3 content in NTIS patients based only on serum T3 levels.

Fasting (or caloric restriction) is a condition that frequently overlaps with NTIS and in many ways also affects TH homeostasis. In humans, caloric restriction or fasting is associated with lower serum T3 levels; serum T4 may be reduced as well and this coexists with normal/low serum TSH (94, 95). However, like what happens to patients with NTIS, the lack of clear signs and/or symptoms of hypothyroidism questions whether tissue T3 content (and/or T3 signaling) is equally reduced.

Studies in rodents in which fasting (short or prolonged) led to a drop in serum T3 of ~35% show variable changes in T3 content among tissues. After a 36h-fasting, the T3 content in the kidneys, hypothalamus, and pituitary was unaltered, but in the liver, it increased by ~30% (44, 96, 97). In prolonged fasting (7 days), the cerebral cortex, cerebellum, and anterior pituitary T3 content dropped by ~35%, but remained unaffected in the skeletal muscle or slightly increased (~8%) in the liver. To our knowledge, there are no similar studies done on humans.

The situation with NTIS and fasting is more complex than hypothyroidism alone because changes in tissue T3 content might not be the only factor when considering T3 signaling. Animal models of NTIS reveal changes in the expression level of TRs and transcriptional coregulators, which could modulate TH signaling on top of the changes in tissue T3 content. In hospitalized patients with septic shock NTIS, the skeletal muscle expression of TRB1 and retinoid X receptors-G (RXRG) was lower and RXRA higher than in control patients (98). In addition, subcutaneous adipose tissue from NTIS patients had lower MCT8, TRB1, TRA1, RXRG, and nuclear receptor corepressor-2 (NCOR2). Thus, in patients with septic shock NTIS, tissue responses are generally oriented to soften TH actions (99). Another example is in the failing human hearts, which exhibit changes in myosin-heavy chain isoforms that resemble the pattern produced in response to hypothyroidism. These changes were linked to a reduction in TRA1 and an increase in TRA2 (which does not bind T3), explaining a local attenuation of TH signaling (100). Notably, this leads to cardiac fetal reprogramming, which is defined by the suppression of adult and re-activation of fetal genetic profile, which may constitute an adaptive process to counteract deleterious events taking place during cardiac remodeling (101). In addition to these thyroid-specific changes, patients with different forms of NTIS exhibit an abnormal release of proinflammatory cytokines and glucocorticoids, which along with the administration of multiple drugs, can potentially interfere with TH signaling (102–104)

Therefore, serum T3 is not a good predictor of tissue T3 or TH signaling in NTIS or fasted patients; and tissue T3 content is not the only determinant of TH action that is affected in patients with NTIS. Changes in multiple levels of the TH action network explain the tissue-specific and multiple regulatory mechanisms that could play a role in mitigating symptoms of hypothyroidism during illnesses.

## Conclusions

Tissue T3 content reflects circulating T3 levels and faithfully informs about TH signaling in most tissues (except for the brain and pituitary gland). The knowledge that in LT4-treated patients serum T3 levels might not be fully restored, raises the concerning possibility that TH signaling in these patients (in most tissues) might also not be fully normalized. Clinicians could consider the potential usefulness of measuring serum T3 levels to monitor the effectiveness of therapy with LT4. Future clinical trials should aim at correlating clinical outcomes with serum T3 levels.

## Author contributions

Both authors researched data for the article, discussed the content, and wrote the manuscript. All authors contributed to the article and approved the submitted version.

## Funding

Supported by DK 15070, DK58538, and DK65055.

## Acknowledgments

The authors are grateful to Profs. Samuel Refetoff and Juan Bernal for kindly providing references that were used in this publication.

## Conflict of interest

AB is a consultant for Synthonics Inc, Allergan Inc, and BLA Technology LLC.

The remaining author declares that the research was conducted in the absence of any commercial or financial relationships that could be construed as a potential conflict of interest.

## Publisher’s note

All claims expressed in this article are solely those of the authors and do not necessarily represent those of their affiliated organizations, or those of the publisher, the editors and the reviewers. Any product that may be evaluated in this article, or claim that may be made by its manufacturer, is not guaranteed or endorsed by the publisher.
